# Implementation of extended scope of practice

**DOI:** 10.1002/jmrs.34

**Published:** 2013-12-03

**Authors:** Jill Harris

**Affiliations:** The Crown Princess Mary Cancer Centre Westmead, NSW, Australia

Re: Monk CM, Wrightson SJ, Smith TN. An exploration of the feasibility of radiation therapist participation in treatment reviews. *J Med Rad Sci* 2013; **60**(3): 100–7.

I am writing in response to the recently published article ‘An exploration of the feasibility of radiation therapist participation in treatment reviews’ by Monk, Wrightson and Smith.^1^

It is particularly interesting that the results of this study do not replicate results of similar studies conducted elsewhere and I note that the authors postulate that these results may be related to local conditions and personalities. I would suggest that this is indeed the case and is in fact a major challenge in introducing advanced/extended scope of practice into the radiation therapy domain. It is not clear from the article whether substantial interprofessional communication occurred prior to the study in order to identify treatment reviews as an area where it was thought appropriate for expansion of the radiation therapist (RT) role to occur.

Radiation therapy is legislatively a medically dominated hierarchy. Radiation oncologists (ROs) take responsibility for the patient's care and indeed, at this stage, legally for any mistake made by RTs during the course of this care. I would suggest that this may be one major contributing factor to the finding that while 80% of RO respondents indicated that they felt RTs were capable of participating in treatment reviews, 0% of the same respondents were willing to delegate responsibility to the RTs.

Gaps in service which RTs are capable of filling will be different in each centre, dependent on staffing, workload and individual priorities, and/or preferences for tasks among the ROs. It would be futile to attempt to assume a task traditionally performed by another profession in a situation where that professional valued that particular task highly.

At Crown Princess Mary Cancer Centre (CPMCC), we have implemented extended scope RT roles in breast, urology, head and neck, and paediatric cancers. Each of these roles has different drivers (identified and agreed gaps in the service) for the creation of the role, differing role descriptors and different expectations of the RTs within the roles. In our experience, constant communication with the ROs is essential in creating the building blocks and trust required to implement this type of role. In each case, the role descriptions were devised through a consultative process where the ROs in charge of that tumour grouping were equal partners in determining the scope of practice. Similarly, in each of these cases, as the ROs have developed a closer relationship with the RTs undertaking these roles, their trust in the RT capabilities has increased and each role has evolved over time, with the RTs now taking far more responsibility than during the initial implementation phase.

To conclude, I would also like to emphasise that, if a gap is identified by the RTs, we should not expect immediate agreement that we should assume additional responsibility. Perseverance to gain agreement of the need is first required, followed by proof of capability. This article shows proof of capability for many patients per week, if agreement could be obtained for the need, I would suggest that a different outcome may be derived.
